# Extraction of Lumbar Spine Motion Using a 3-IMU Wearable Cluster

**DOI:** 10.3390/s23010182

**Published:** 2022-12-24

**Authors:** Kee S. Moon, Sara P. Gombatto, Kim Phan, Yusuf Ozturk

**Affiliations:** 1 Mechanical Engineering, College of Engineering, San Diego State University, San Diego, CA 92182, USA; 2 Doctor of Physical Therapy Program, School of Exercise and Nutritional Sciences, San Diego State University, San Diego, CA 92182, USA; 3 Electrical and Computer Engineering, College of Engineering, San Diego State University, San Diego, CA 92182, USA

**Keywords:** wearable biomedical sensors, wireless network, medical equipment, multi-sensor fusion, inertial measurement unit, robotic simulator, low back pain, body kinematics, body area network (BAN)

## Abstract

Spine movement is a daily activity that can indicate health status changes, including low back pain (LBP) problems. Repetitious and continuous movement on the spine and incorrect postures during daily functional activities may lead to the potential development and persistence of LBP problems. Therefore, monitoring of posture and movement is essential when designing LBP interventions. Typically, LBP diagnosis is facilitated by monitoring upper body posture and movement impairments, particularly during functional activities using body motion sensors. This study presents a fully wireless multi-sensor cluster system to monitor spine movements. The study suggests an attempt to develop a new method to monitor the lumbopelvic movements of interest selectively. In addition, the research employs a custom-designed robotic lumbar spine simulator to generate the ideal lumbopelvic posture and movements for reference sensor data. The mechanical motion templates provide an automated sensor pattern recognition mechanism for diagnosing the LBP.

## 1. Introduction

Low back pain (LBP) is one of the leading causes of disability that affects up to 80% of people and is second only to respiratory illnesses with regard to days of lost work [1,2]. Lumbopelvic movements have been identified as significant risk factors for developing LBP and indicators of LBP rehabilitation progress [3,4]. Although injury to mainly the intervertebral disc is one commonly known source of the LBP development, we can identify the LBP symptoms by monitoring the performance of the same lumbopelvic movement, based on speed, angle range (range of motion), acceleration, and intensity to identify movement differences between individuals with and without LBP. Therefore, these features can be used to characterize each lumbopelvic movement for individuals with and without LBP [5,6]. Research has shown that repetitive and prolonged stresses on the spine associated with a person’s posture and movement can be a source of potential LBP problems [7]. Therefore, it is essential to assess posture and movement for a patient with LBP in the clinic. It is also crucial to evaluate posture and movement throughout the day during functional activities at home and work.

Three-dimensional motion capture systems have been used to examine the posture and movement associated with LBP that might contribute to increased stress on tissues in people with LBP [8]. Others have recognized the importance of quantifying kinematics during more functional tasks related to LBP. Body motions associated with the spine during everyday activities can manifest as the stresses applied to low back [9]. In many cases, the posture and movement measurements at the laboratory provide limited information about the person’s posture and movement due to time and location restrictions. Therefore, we would need hours of ecological monitoring throughout the day to measure the cumulative effects of stresses on the spine associated with prolonged postures and repeated movements. Further, the measurements of daily posture and movement can provide more comprehensive information about the contributions of the person’s activity to the development and persistence of an LBP problem. Providing feedback on posture and movement throughout the day can help people develop greater awareness of their incorrect posture and movements, thus improving their voluntary control [10].

There is a need for an unobtrusive small wireless sensor system to quantify the spine posture and movement during everyday activities that can measure the daily stresses associated with posture and movement concerning the anatomical angles, such as the motions of back in sagittal (SA), frontal (FR) or transverse (TR) planes. In particular, recent mobile and wireless technology advances especially offer an opportunity to collect physiological signals and body movement data remotely. Due to this advancement, some sensor systems, including vision-based systems and inertial measurement unit (IMU)-based systems that can monitor fine granularity angular body movements have emerged in the market [11,12].

One common characteristic of these systems is the ability to compute the angle by sensing coordinates of different points on a human body in a three-dimensional space. These data can also be used to calculate the angular velocity, acceleration, and direction. Therefore, they require the application of multiple IMUs to provide sensing coordinates of different points on a human body in a three-dimensional space [13]. The inertial gyro sensors consist of three axes based on the micro-machined structures on a silicon wafer. Therefore, inertial sensors usually require aligning the sensor axes with the underlying anatomical axes to be related to the body motions. Emerging multi-IMU alignment methods with a human joint model allows overcoming the IMU sensor misalignment issue [14]. This study presents the feasibility of using three IMU sensors to detect three-dimensional lumbopelvic movements, including flexion, extension, pelvic tilt, lateral flexion, and rotation to the body frame to address the methodological challenges of inertial sensor applications for LBP [15]. Machine learning techniques have been applied to automate the extraction of the specific postures or motions to monitor the LBP [16].

This paper introduces a method to measure and analyze the patterns of the sensor cluster outputs for detecting the lumbopelvic movements to a moving coordinate system. The cluster uses a network of three IMU sensors placed at a triangular location on a lumbopelvic area to extract the relative motion between them while in action. These sensors are custom-made at San Diego State University [17,18]. The small form factor, wearable wireless sensor, utilizes Bluetooth for connectivity. The sensor causes minimal discomfort while wirelessly monitoring limb-segment movement for prolonged durations due to the long battery life of the sensors. In addition, the wireless sensor does not impose restrictions on location for data collection as data can be collected at any location, including outdoor environment or in the comfort of home. In summary, the paper describes a novel approach for detecting the specific lumbopelvic movements of interest with a wireless network system. In addition, we introduce a method to generate the lumbopelvic reference movements using a robotic spine simulator. Finally, we compare the reference lumbopelvic movement templates with human data to extract and detect the specific lumbopelvic activities.

## 2. Lumbopelvic Kinematics

One of the primary components of low back pain (LBP) physical examination is monitoring the lumbopelvic movement and posture [19,20,21]. Evaluation of lumbopelvic movement can include basic kinematic assessments, such as range of motion (ROM) and posture, or complex kinematic patterns, functional activities such as walking or lifting.

**Figure 1:**
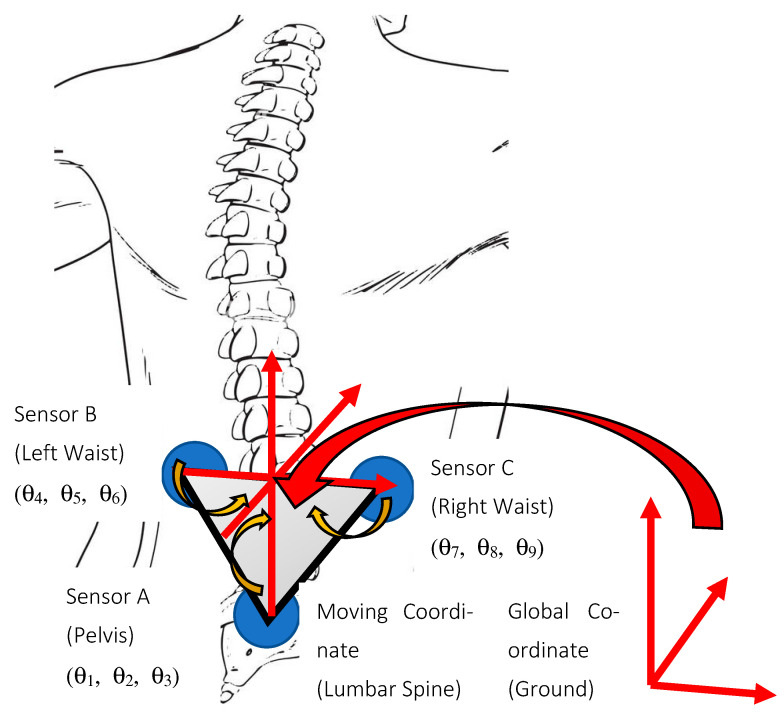
Approach to calculate relative motion to the ideal position and transform the coordinate system of the lumbar sensors (B and C) with the pelvis sensor (A).

Our body moves in three planes of motion. Ideally, to evaluate the lumbopelvic movement in sagittal, frontal, and transverse planes, the wearable sensors need to be aligned perfectly with respect to the three planes so that the angular motion of each sensor will be measured in the same plane. First, however, each sensor needs to be calibrated to the planes of motion because of the body contours. In this study, a network of three IMU wearables is used to form a cluster to evaluate the angular motion of the lumbar spine, which is displayed in Figure 1. Three IMU sensors are used to compensate for sensor installation imperfections. The sensors are attached to skin rather than the bone, therefore they may have a drift relative to the spine. To eliminate the impact of the drift with respect to the spine, two sensors are attached, one on each side of the spine. The motion of these two sensors placed on each side of the spine with respect to the reference sensor placed on pelvis is used to filter out the effect of skin movements. When motion data is transmitted to the host application, the motion of each sensor node is reported to its axis. When the sensors are not perfectly aligned, the two sensors will be reporting movement in two different coordinate systems, resulting in measurement errors.

In this study, we apply a coordinate transformation to align the coordinate systems of the sensors. Further, we calculate the relative motion of the lumbar spine in combination with the pelvis. As shown in Figure 1, the lumbar spine contains five intervertebral discs situated between the vertebral bodies. Thus, as a kinematic manipulator, the lumbar spine has multiple physical links and joints, each having three rotational joints. Consider a simplified kinematic model of physical joint A at the pelvis (pelvic joint), and a physical joint B, situated on the lumbar spine. The physical joint has three degrees of freedom (DOF) imparting a robot system. Sensor A is placed at the end of the pelvis and provides the orientation of the pelvis to the base frame (i.e., ground). Sensor B is situated at the end of the lumbar spine, and it provides the orientation of the lumbar spine to the base frame (ground). Note that the directions of both sensors are maintained the same.

Three-dimensional kinematics of the pitch (sagittal-SA), yaw (transverse-TR), and roll (frontal-FR) angles of the three IMUs are simultaneously measured and recorded wirelessly into a PC. The following calculation procedures are implemented to convert the three sets of the pitch (SA), yaw (TR), and roll (FR) angles of the IMUs into the anatomical body planes that are hypothetical geometric planes used to describe the location or direction of bodily structures:

(a) Define the transformation matrices $T_{A\;\left(original\right)}^{0}$, $T_{B\;\left(original\right)}^{0}$, and $T_{C\;\left(original\right)}^{0}$ as the mapping of the sensors A, B, and C in the base frame. For the sensor orientation matrices to have the same coordinate at the first starting position, the calibrated matrix of sensor A ($T_{A}^{0}$) is calculated by
  (1) $$T_{A\;\left(inv\right)}^{0}={\left[T_{A\;\left(original\right)}^{0}\left(at\;initial\;position\right)\right]}^{-1},$$
  (2) $$T_{A}^{0}=T_{A\;\left(original\right)}^{0}\;T_{A\;\left(inv\right)}^{0}.$$
  Similarly, the calibrated rotation matrix of sensor B ($T_{B}^{0}$) and sensor C ($T_{C}^{0}$) are provided by
  (3) $$T_{B\;\left(inv\right)}^{0}={\left[T_{B\;\left(original\right)}^{0}\left(at\;initial\;position\right)\right]}^{-1},$$
  (4) $$T_{B}^{0}=T_{B\;\left(original\right)}^{0}\;T_{B\;\left(inv\right)}^{0},$$
  (5) $$T_{C\;\left(inv\right)}^{0}={\left[T_{C\;\left(original\right)}^{0}\left(at\;initial\;position\right)\right]}^{-1},$$
  (6) $$T_{C}^{0}=T_{C\;\left(original\right)}^{0}\;T_{C\;\left(inv\right)}^{0}.$$

Note that only the inverse of the first matrices of $T_{A\;\left(original\right)}^{0}$, $T_{B\;\left(original\right)}^{0}$ and $T_{C\;\left(original\right)}^{0}$ are taken so that the first starting matrices of initialized $T_{A}^{0}$, $T_{B}^{0}$ and $T_{C}^{0}$ always become an identity matrix.

(b) Define $T_{B}^{A}$ as the mapping of sensor B in the sensor A frame. In addition, $T_{C}^{B}$ and $T_{C}^{A}$ as they can be described as follows based on the Denavit and Hartenberg (D–H) convention. The global homogeneous transformation matrix $T_{0}^{G}$ can be assumed to be an identity matrix. For example, the mapping matrix of sensor B in the sensor A frame can be calculated by defining the forward kinematic equation to obtain the three sensor orientations to the base frame (frame 0).
  (7) $$T_{B}^{G}=T_{0}^{G}\;T_{A}^{0}\;T_{B}^{A}.$$
  Rearranging the above equation to obtain the orientation of sensor B with respect to sensors A provides
  (8) $$\;T_{B}^{A}={\left(T_{A}^{0}\right)}^{-1}\;{\left(T_{0}^{G}\right)}^{-1}\;T_{B}^{0},\;$$
  where $T_{B}^{0}$ is the calibrated version of $T_{B\;\left(original\right)}^{0}$.
  In addition, the mapping matrix of the sensor C in sensor A frame is
  (9) $$\;T_{C}^{A}={\left(T_{A}^{0}\right)}^{-1}\;{\left(T_{0}^{G}\right)}^{-1}\;T_{C}^{0},$$
  where $T_{C}^{0}$ is the calibrated version of $T_{C\;\left(original\right)}^{0}$.
(c) Finally, by applying inverse kinematics to $T_{A}^{0}$, $T_{B}^{A}$, and $T_{C}^{A}$, the 3-D pelvis joint angles $\theta_{1}$, $\theta_{2}$, $\theta_{3}$, the 3-D lumbar spine joint angles $\theta_{4}$, $\theta_{5}$, $\theta_{6}\;,\;\theta_{7}$, $\theta_{8}$, and $\theta_{9}$ (Figure 1) can by calculated in same the anatomical body planes.

## 3. Inertial Measurement Unit System

**Figure 2:**
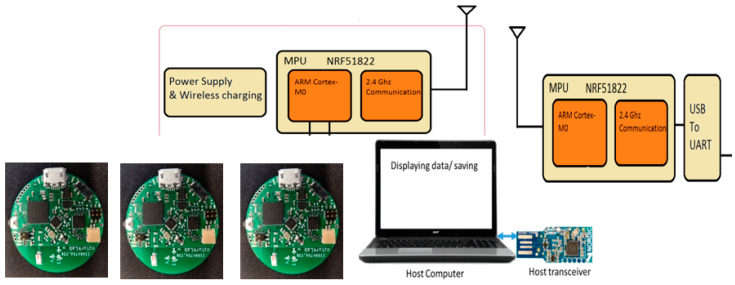
A custom designed IMU network to record lumbopelvic movements: the sensor system diagram and the manufactured wireless IMU sensor circuit that is in the size of a diameter of 23 mm.

The advancement of the IMU sensor technology introduces a new way of automated monitoring and diagnosing human motion patterns and a computer-aided method of analysis, graphic visualization, storage, and archiving [22]. Figure 2 describes the IMU integrated sensor cluster system to detect lumbar spine activity without wiring between the sensor nodes at different locations. This paper describes the method to acquire and analyze the IMU data to detect the lumbopelvic kinematic motion. The main system configuration is a cluster of IMU sensors to create a human trunk network of multiplications. Inertial measurement units provide angular turning rates and three-axis acceleration data using a cubical magnetically suspended sensor mass.

This paper presents a novel way to remotely monitor three-dimensional lumbopelvic movements using a three-IMU cluster system to detect the LBP-related lumbar spine motion changes [23]. The IMU uses a combination of a gyroscope and an accelerometer to measure the motion of the mass. The gyroscope provides the angular turning rates while the accelerometers provide linear motion. The motion data from the IMU is reported in the form of quaternions. For this research, rotation matrices are obtained from the quaternions.

The study uses Bluetooth Low Energy (BLE-5.0) for short-range wireless transmission, and a laptop computer acts as a host receiver for the data transmitted. A MATLAB program involving mathematical calculations provides the analysis of the sensor data. The wearable sensor assembly has an MPU-9250 (InvenSense) chip with a 5°/h stability at the 20 Hz sampling rate. The wearable node uses a Nordic Semiconductor nRF51822 communication system on a chip (SoC) for a communication module. The SoC supports a maximum data transmission rate of 2 Mbps. An onboard Li-Polymer charge management controller MCP73831 recharges the sensor module [17].

## 4. Robotic Lumbopelvic Simulator

**Figure 3:**
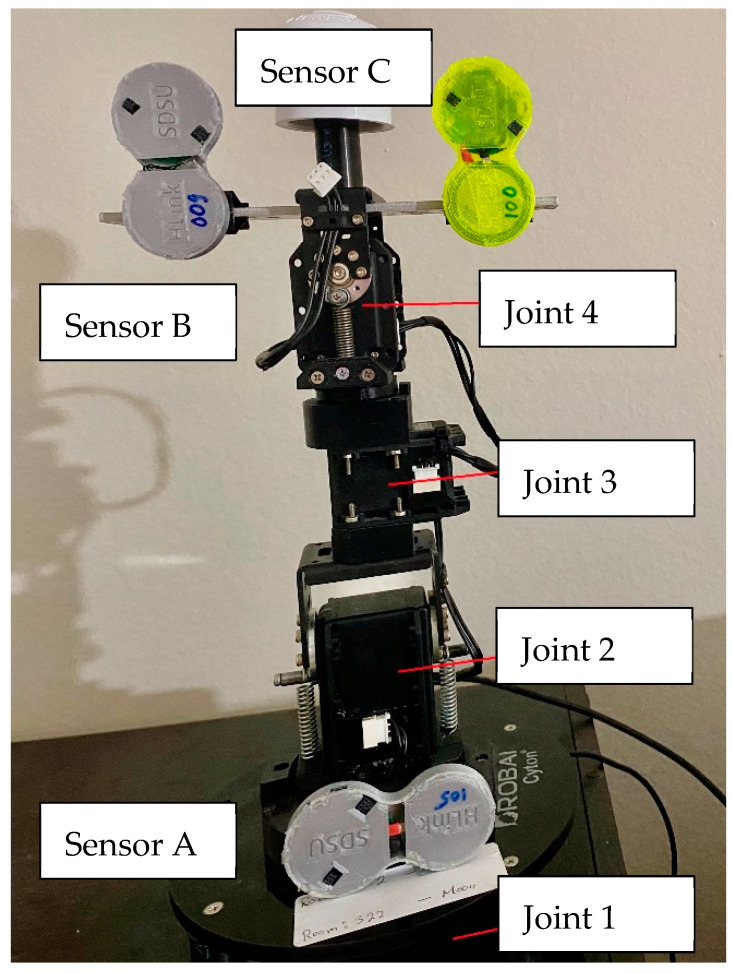
A four-degree-of-freedom (DOF) robotic spine simulator which consists of four links is developed using four servo motors. The wearable sensor cluster communicates wirelessly using Bluetooth low-energy (BLE) technology.

This section describes the study to build a robotic spine motion simulator to reproduce lumbopelvic mechanics. It provides a unique way of estimating the IMU sensor signals using four degrees of rotary joints and links set. The robotic simulation method introduces a new IMU signal processing method that saves computation time and scalable machine learning algorithms. Using new statistical feature extraction, the new approach can integrate raw sensor data with varying reliability and sensitivity. The proposed method is suitable for daily use and to engage in digitally interactive low back monitoring. A four-DOF robotic spine simulator which consists of four links is developed using four servo motors (Dynamixel MX, Robotis) as shown in Figure 3.

MATLAB-based simulation programs were designed to conduct the kinematics of the robot simulator in this study. In this study, the base (first link) is fixed. The motors are set on the rotational joints and allow the user to change the angular values of each joint to simulate the lumbopelvic movements. The goal is to find the ideal (or template) sensor signals from the IMUs attached between the first and last links (end of the lumbar spine). The user can write a program in MATLAB to move the simulator to the desired position for recording corresponding sensor signals.

The wearable sensor cluster with the three IMU nodes communicates wirelessly using Bluetooth low-energy technology, as shown in Figure 3. The system acquires real-time signals from multiple sensors simultaneously. The individual IMU sensor receives the orientation signals and supplies them to the analog front end. After that, the signal is transmitted wirelessly to a computer.

## 5. Experimental Procedure and Results

LBP is one of the widespread musculoskeletal pain syndromes worldwide [21]. For example, approximately 85% of patients with LBP patients have no obvious pathoanatomic/radiologic defects. Further, the analysis of spinal postures and movements is essential to understanding the easing and aggravating factors of LBP. For example, one of the most common mechanical causes of lumbar injury is the flexion movement of the spine, an everyday movement in daily activities. Therefore, knowledge of biomechanics and their preventive strategy of lumbar and hip posture and coordination during flexion can help manage LBP [24,25,26].

In this study, a wearable sensor cluster was designed and implemented using three custom-designed IMUs [17,18], as shown in Figure 2 and Figure 3. An experimental protocol was followed while collecting the data in the described sensor placement. All the experiments were performed in the standing posture. The conducted experiments are presented in Table 1. Experiment I is the standard case showing flexion and extension pattern with bend–extend trunk motion in the table. The flexion and extension movements occur within the sagittal plane and involve anterior or posterior activities of the body. The flexion experiment is an anterior (forward) bending of the trunk, while extension involves a posterior-directed motion, such as bending backward. Experiment II includes a rotation pattern that is the twisting movement of the upper body toward the right or left side. Finally, Experiment III has a lateral flexion pattern that bends the upper body toward the right or left side.

In this study, we conducted a non-clinical experiment on the robotic lumbopelvic simulator and a single person (i.e., an author). The experiments were performed multiple times to ensure the repeatability of the results. Since only a single individual performed the feasibility test, the sampling size was not statistically significance could not be determined. However, for proof-of-concept purposes, the test result was encouraging.

**Figure 4:**
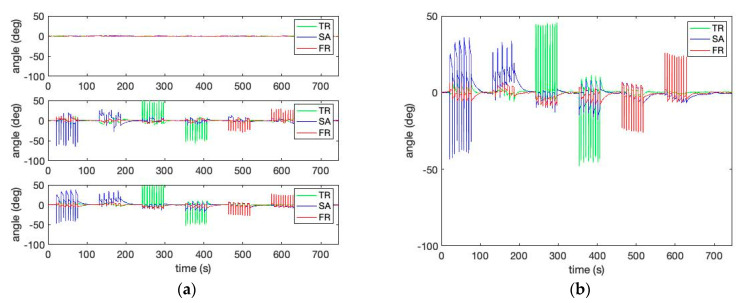
(**a**) Three-dimensional kinematics of the pitch (SA), yaw (TR), and roll (FR) angles of the three IMUs; (**b**) the combined three-dimensional kinematics of the pitch (SA), yaw (TR), and roll (FR) angles from the three IMU cluster system.

Figure 4 shows the three kinds of lumbopelvic patterns described in Table 1. The figure shows the relative curves of the lumbar spine angles during the trunk motion patterns of Table 1 in the sagittal, frontal, and transverse planes. In addition, the figure shows that the three IMU cluster system provides a better estimation of the spine orientations than the individual IMUs.

This study presents a new method of using a signature matrix-based pattern classification [27]. The method generates three-dimensional signature matrix patterns from the calculated lumbopelvic angles. For example, we can set up the trunk motion classification templates from a well-planned series of robotic simulator experiments to capture the characteristic (or signature) behavior of the lumbopelvic kinematic motions. These signature matrix numerical values reflect the compressed characteristics of the trunk motion patterns using a probability map.

The signature matrix elements indicate a calculated probability that the three-dimensional angles fall into a specific classification criterion. For example, to calculate the probability map, a frequency count can be calculated from matching three-dimensional angles (i.e., pitch, yaw, and roll) for a previously classified criterion or zone of the range of motion (ROM). Thus, a signature matrix element $sig_{ijk}$ can be calculated by

(10) $$sig_{ijk}=P\left[\left(Pitch\;within\;\mathrm{ROM}\;i\;\right)\cap^{\;}\left(Yaw\;within\;\mathrm{ROM}\;j\;\right)\cap^{\;}\left(Roll\;within\;\mathrm{ROM}\;k\;\right)\right].$$

**Figure 5:**
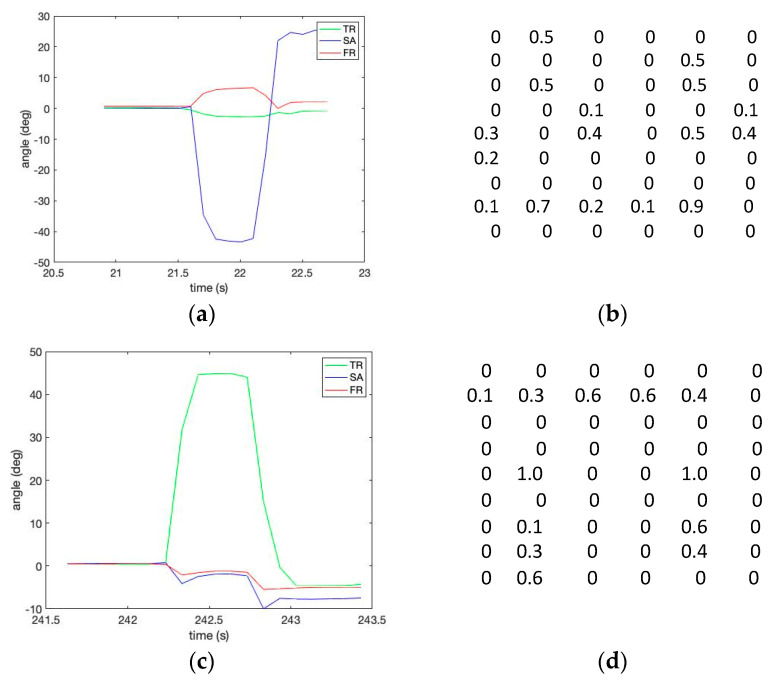
The template signature matrix data windows in the sensor signals: (**a**) the template angles for Experiment I—sagittal forward flexion 60°; (**b**) the template matrix for Experiment I—sagittal forward flexion 60°; (**c**) the template angles for Experiment II—transverse right rotation 50°; (**d**) the template matrix for Experiment II—transverse right rotation 50°.

In this study, a [3 × 3 × 3] three-dimensional signature matrix was rearranged as a two-dimensional probability map with a [6 × 9] matrix size for the convenience of visualization, as shown in Figure 5. The data to calculate a matrix were collected in a subgroup size of 40 sampling points in a two-second window. Figure 5 shows the 6 × 9 signature matrix formation used to reflect the compressed characteristics per the given trunk motion pattern described in Table 1.

An offline sensor data assessment was conducted by comparing the measured signature matrix patterns (i.e., targets) with the stored template patterns. The squared difference between the template pixel and the measured pixel is provided by

(11) $$d_{ijk}^{k}={\left(sig_{ijk}^{T}-sig_{ijk}^{k}\right)}^{2}.$$

The sum of squared differences at the sampling period *k* can be calculated to observe the errors between the pre-stored template signature matrices and the measured matrices.

(12) $$E_{ijk}^{k}=\sum_{i=1}^{N}\sum_{j=1}^{N}\;\;\;\sum_{k=1}^{N}{\left(sig_{ijk}^{T}-sig_{ijk}^{k}\right)}^{2}.$$

**Figure 6:**
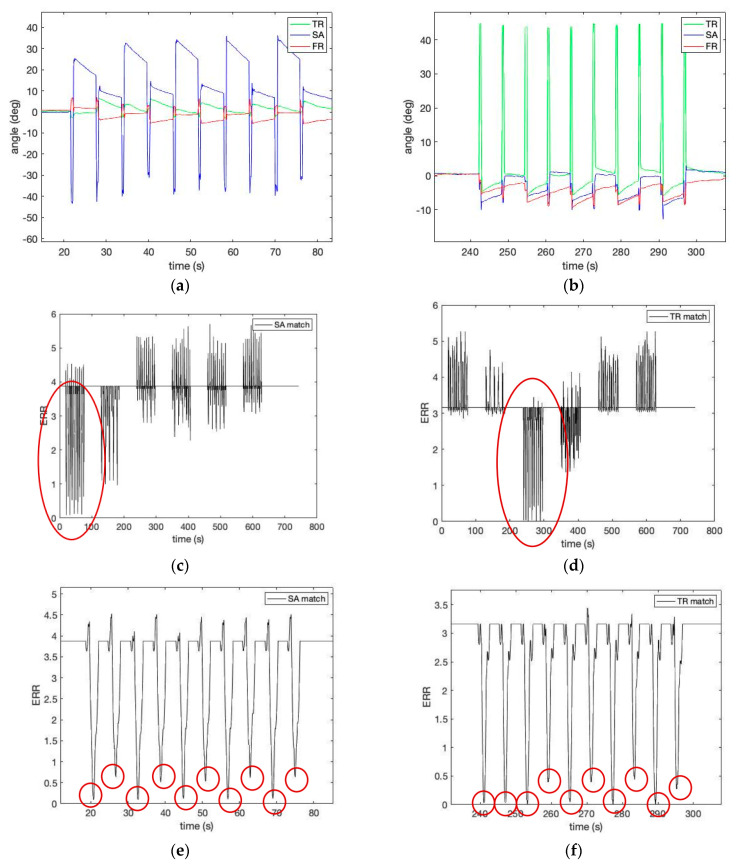
The estimated robot simulator motion pattern: (**a**) the measured angles for Experiment I—sagittal forward flexion 60°; (**b**) the measured angles for Experiment II—transverse right rotation 50°; (**c**) the matching error level from Experiments I, II, and III calculated by the template matrix of sagittal forward flexion 60°; (**d**) the matching error level from Experiments I, II, and III calculated by the template matrix of transverse right rotation 50°; (**e**) the matching error level from Experiment I calculated by the template matrix of sagittal forward flexion 60°. Red circles demonstrate low matching error.; (**f**) the matching error level from Experiment I calculated by the template matrix of transverse right rotation 50°. Red circles demonstrate low matching error.

Figure 6 shows the estimated robot simulator motion pattern in Table 1 by calculating Equation (12) using the template matrix provided in Figure 5. Please note a low matching error level (shown as a red circle). The robotic spine simulator experiment with the three-IMU network system showed promising results in measuring three-dimensional joint angles of lumbopelvic motion. In addition, the proposed method to calculate the three-dimensional relative angles of the lumbar spine movements to the pelvic joint using a cluster network of IMUs is valid.

**Figure 7:**
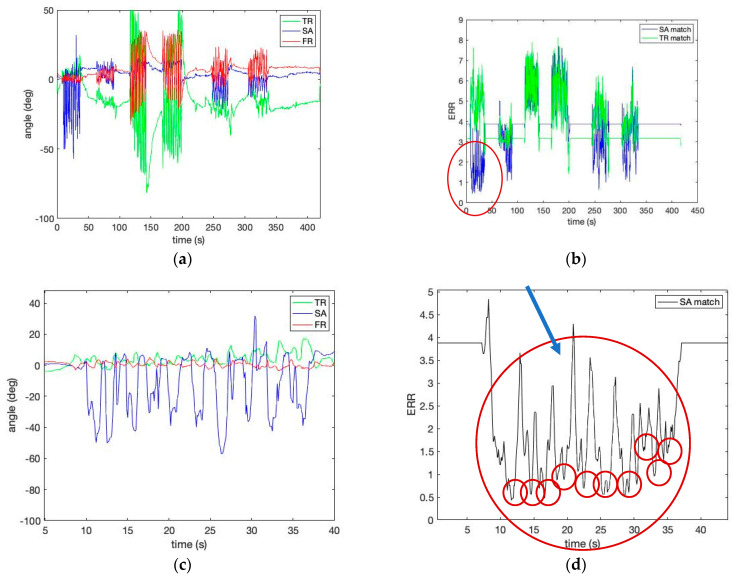
The estimated human motion pattern: (**a**) the measured angles for Experiment I, II, III; (**b**) the matching error level from Experiments I, II, and III calculated by the template matrices of sagittal forward flexion 60° and transverse right rotation 50°; (**c**) the measured angles for Experiment I—sagittal forward flexion 60°; (**d**) the matching error level from Experiment I (the red circled graph of (**b**)) calculated by the template matrix of sagittal forward flexion 60°. Red circles demonstrate low matching error.

Figure 7 shows the estimated human motion pattern in Table 1 by calculating Equation (12) using the template matrix obtained from the robotic simulator’s motion provided in Figure 5. Please note that the speed of human movement is slower than that of the robot simulator, although the number of repetitions is the same (i.e., 10 repetitions per pattern). In identifying the lumbopelvic kinematic motions from a human study, Figure 7 shows evidence of the ROM angle changes between the ideal (i.e., template) and the person (i.e., target) during flexion motion. In addition, Figure 7 shows that the lumbopelvic angles are mixed in all directions. Understandably, human lumbopelvic coordination would be difficult to conduct in one-directional movement [28].

## 6. Discussions

Although the preliminary experimental results of the human lumbopelvic motion showed reasonably compatible accuracy in all three directions (i.e., sagittal, frontal, transverse), the IMU-based motion measurement method is generally known to have lower accuracy than optical camera-based methods [29]. The typical IMU drifting due to the error accumulation in the gyroscope’s integral value was still a significant problem. Further, there were errors from the inherited nature of inertia-based sensors, such as abrupt changes at peaks or valleys during motion. When considering this individual variability in spine alignment, using a body coordinate system can help adjust for alignment variation or deformity to obtain accurate values for any individual. Developing a body coordinate system to account for variation in mounting alignment across individuals is vital for the spine and other body regions. Regardless of the body region, considering individual variability in resting alignment and placement of the sensors when calculating LBP measures is necessary for valid interpretation of the data. However, having three IMUs placed on both sides of the trunk seems to help obtain a more meaningful measurement of body movement.

The IMU-based motion measurement can introduce significant artifacts during daily activities such as walking. On the other hand, the proposed method can potentially identify specific lumbopelvic kinematic motions of interest. In future studies, we plan to develop more reference templates representing the three-dimensional lumbopelvic kinematic patterns. The larger set of templates will be suitable for training various machine learning methods to estimate low back movement.

Therefore, in this study, we introduce two new indices to estimate the characteristic changes of the IMU signal for our wearable. We define the rate (*Rs*) as the number of low-frequency repetitions of a specific lumbar spine motion pattern *s* per minute as shown in Figure 6e,f and Figure 7d. Each successive time difference between the two repetitions *x_i_* is calculated as

(13) $$x_{i}=t_{n}-t_{n-1},$$

where *t_n_* denotes the interval of the *n*-th repetition in the signal.

(14) $$Rs={\left[\left(\sum_{i=1,N}x_{i}\right)/N\right]}^{-1},$$

where *N* denotes the total number of the repetition intervals in the signal per unit period.

Further, the repetition interval variability (*Vs*) is defined as the root mean square of successive differences between the repetitions that are obtained by first calculating each successive time difference between the repetitions (*x_i_*) from Equation (13). Then, each of the values of *x_i_* is squared and averaged. We expect that the *Vs* reflects the time-domain index used to estimate the short-term IMU signal changes.

(15) $$Vs=\sqrt{\frac{1}{N}\sum_{i=1,N}x_{i}^{2}}.$$

In addition, the *Vs* can be an index to help us monitor LBP during daily motion. The *Vs* indexes lumbar spine movement function and could be generated by LBP, although it will require more investigation in the future. Only a single individual performed the human subject test; thus, the sampling size was not statistically significance could not be determined. The experimental results are not statistically tested for clinical purposes. It will require further clinical research in the future.

## 7. Conclusions

We proposed a wearable device network and the associated analysis method to enable remote LBP monitoring, which is one of the fastest-growing telemetry technologies. Monitoring and analyzing lumbopelvic movement data can provide essential LBP information for the users and the public healthcare communities. For example, the assessment ability of cyclical movement patterns based on biomedical kinematics may allow early recognition of the LBP signs of illness. We proposed a three-IMU cluster system to address the limitations of using a single IMU-based LBP measurement to measure and monitor lumbopelvic posture and movement patterns. The work contributes to classifying the characteristic lumbopelvic movement patterns of the pelvis and the spine joints. The study conducted the following steps to recognize the specific movement patterns of interest:

- Calculation of 3D angles of the hip and spine in the sagittal, frontal, and transverse planes from the three IMU sensor network system;
- Conversion of the 3D lumbopelvic movement angles into a train of signature matrices;
- Estimation of the matching error level from the predetermined template motion obtained from the robotic simulator.

The work contributes to the IMU signal analysis by introducing a new method suitable for establishing the patterns of different LBP movements. Furthermore, the proposed processes contribute in many ways to the state-of-the-art identification and modeling of the IMU signal analysis for lumbopelvic motion applications. For example, the approach could increase the pattern classification capability to make an intelligent machine learning system. However, please note that the authors collected human subject data from only the first author due to the challenges of receiving approval of the institutional review board (IRB) to conduct human subject research during the COVID-19 pandemic. We will obtain an IRB approval to collect data from a larger set of subjects and apply machine learning such as (SVM) or (LSTM) in a future study for template matching.

## Figures and Tables

**Table 1:** The experiment designs.

Experiment	Trunk Motion Pattern	Angular Motion
I-Sagittal	Forward Flexion	60°
	Backward Extension	20°
II-Transverse	Right Rotation	50°
	Left Rotation	50°
III-Frontal	Right Lateral Flexion	30°
	Left Lateral Flexion	30°
